# Non-Hermitian Dynamics in Three-Level Systems: A Perturbative Approach for Time-Dependent Hamiltonians

**DOI:** 10.3390/e28030268

**Published:** 2026-02-28

**Authors:** Guixiang La, Yexin Li, Gongping Zheng

**Affiliations:** College of Physics and Electronic Information Engineering, Qinghai Normal University, Xining 810016, China; 202339331039@stu.qhnu.edu.cn (G.L.); 18954038824@163.com (Y.L.)

**Keywords:** non-Hermitian physics, non-Hermitian dynamics, perturbation theory, asymmetry, biorthogonal basis

## Abstract

The conventional time-dependent perturbation theory in quantum mechanics is established within the framework of Hermitian Hamiltonians, applicable for describing quantum transitions and associated energy level responses in such systems. However, this theory has fundamental limitations when applied to non-Hermitian systems. Consequently, researchers have systematically extended time-dependent perturbation theory to non-Hermitian systems, establishing a corresponding mature framework. Building on this foundation, this study extends the theory to investigate the transition dynamics induced by non-Hermitian interactions in non-Hermitian Hamiltonian systems. We employ a biorthogonal basis representation for a three-level non-Hermitian system. This work investigates a system comprising an unperturbed static non-Hermitian Hamiltonian and a periodically driven time-dependent perturbation Hamiltonian. Taking the three-level system as a concrete example, we combine analytical methods with numerical simulations to solve and analyze its dynamical evolution equations. These complementary approaches reveal that when system parameters complete a full cycle around an exceptional point, the transitional behavior exhibits specific evolutionary patterns. In this system, quantum transition probabilities exhibit significant asymmetry and non-conservation that depend on the initial and final states, revealing inherent directional characteristics in the dynamical process. Furthermore, for a three-level, periodically driven non-Hermitian system with time-dependent perturbations, this asymmetry is even more pronounced, manifesting as a distinct disparity between forward and reverse transition probabilities. The periodic driving actively amplifies the asymmetry in the transition process. By designing the perturbation spectrum, selective manipulation of specific quantum states can be achieved. Moreover, transition probabilities can be significantly enhanced under resonance conditions, while non-Hermiticity further breaks the system’s inherent symmetry, leading to substantial amplification of transitions in a single direction. By precisely tuning the drive frequency, interactions between specific coupling channels can be selectively enhanced or suppressed. The amplification of channel asymmetry by non-Hermitian properties provides a novel mechanism for directional control of quantum states and opens new pathways for realizing related quantum technologies.

## 1. Introduction

In recent years, research on non-Hermitian physics has attracted widespread attention [1,2,3]. In classical quantum mechanics, Hermitian operators require their eigenvalues to be real numbers, ensuring the unitarity and observability of isolated systems [4]. However, non-conservative processes such as energy dissipation or particle exchange are ubiquitous in actual physical systems. Therefore, such systems are open systems [5,6,7]. The non-unitary dynamics of these systems cannot be adequately described within the framework of conventional perturbation theory, which is based on unitary evolution, thereby necessitating the adoption of a non-Hermitian framework.

Currently, several parallel approaches have emerged for developing non-Hermitian descriptions of open quantum systems. Non-Hermitian Hamiltonians directly characterize gain and loss through the introduction of complex potentials, thereby yielding complex eigenvalues. Singularities, known as exceptional points (EPs), may arise at which both eigenvalues and eigenstates become simultaneously degenerate [8,9]. The quantum quasinormal mode theory provides a systematic non-Hermitian mode-expansion framework for dissipative systems, such as cavity quantum electrodynamics [10]. The Lindblad master equation, derived from the evolution of the density matrix, provides an effective description of dissipative effects and coherent coupling processes in topological systems and quantum technologies [11]. Collectively, the aforementioned theoretical tools provide a formal foundation for the non-Hermitian description of open quantum systems.

The non-unitary dynamics and non-conservation of probability inherent in non-Hermitian systems necessitate the development of specialized mathematical tools, such as perturbation theory tailored for non-Hermitian Hamiltonians. This framework reveals a series of physical phenomena distinct from those in Hermitian systems. Near exceptional points (EPs), both the energy spectrum and the eigenstates undergo significant restructuring [12,13,14,15,16,17,18]. PT symmetry enables real spectra and symmetry-breaking phase transitions in gain–loss-balanced systems [19,20,21,22]; non-Hermitian Floquet theory [23,24,25,26] reveals the mechanism by which periodic driving controls non-equilibrium topological phases—specifically, coherent optical driving can induce Floquet topological quantum states in semiconductors [27]. The non-Hermitian non-local theory successfully characterizes the robust skin effect and bulk-edge correspondence in driving systems [28]. Furthermore, the non-Hermitian skin effect [29,30,31] localizes numerous eigenstates near the boundary. Recent studies have revealed that impurities can induce a reverse skin effect and linear localized modes, thereby significantly expanding the degrees of freedom for controlling this phenomenon [32]. The aforementioned non-Hermitian phenomena have been experimentally verified in platforms including optics [33,34,35,36], acoustics [37,38,39], and electromagnetics [40,41,42,43], thereby stimulating research on the non-equilibrium control of topological states of matter. More significantly, this framework has recently demonstrated potential for applications in quantum technologies. The exceptional-point phase transition in dissipative environments reveals a dynamical phase transition pathway distinct from conventional singularities, offering a new principle for achieving high-sensitivity sensing [44]. Recent advances in non-Hermitian theory for open systems also provide formal tools for decoherence suppression, with applications in areas such as quantum computing [45]. Such interdisciplinary progress indicates that non-Hermitian physics is evolving from fundamental theory to engineering-oriented exploration in the control of open quantum systems.

Notably, the non-Hermitian framework has demonstrated highly controllable experimental advantages in quantum simulation research, providing an ideal platform for exploring novel phenomena such as non-Hermitian topological phase transitions, exceptional-point physics, and non-Hermitian quantum walks. Relevant experiments have been extensively conducted in artificial quantum systems, including photonic lattices, waveguide arrays, and superconducting quantum circuits [46,47,48]. At the level of quantum technology applications, non-Hermitian systems have demonstrated unique potential to surpass the standard quantum limit. The extreme parametric sensitivity of exceptional points and the pronounced amplification of boundary states arising from the non-Hermitian skin effect open new avenues for the development of next-generation ultra-precision quantum sensors [49,50]. Whether in the in-depth exploration of fundamental quantum simulation or the eventual realization of quantum sensing technologies, the core challenge lies in a thorough understanding and precise control of the dynamical evolution of non-Hermitian systems [51,52]. Consequently, developing a time-dependent perturbation theory applicable to non-Hermitian systems and systematically investigating their dynamical behavior have become a crucial scientific step bridging the static properties of non-Hermitian systems and their quantum applications. To quantitatively describe such dynamical processes, a response theory extending beyond the conventional framework is required. Traditional Hermitian linear response theory is primarily applicable to equilibrium correlations in closed systems and is inadequate for addressing dissipation and noise in open systems, nor can it distinguish between topologically equivalent yet dynamically distinct quantum states. By systematically incorporating dissipation mechanisms, non-Hermitian response theory provides a more general non-equilibrium dynamical framework for describing realistic open systems [53].

In recent years, non-Hermitian dynamics have continued to attract extensive research attention [54,55,56,57,58,59,60]. The development of non-Hermitian dynamical theories typically begins with low-dimensional models. As the most fundamental two-level systems in non-Hermitian physics, their parameter space hosts only isolated second-order exceptional points (EPs), accompanied by simple and well-defined eigenstate exchange processes. Research in this direction has become relatively mature [61,62,63,64,65,66,67]. However, owing to the presence of only a single coupling channel in two-level systems, non-Hermiticity can merely modulate the strength of this channel. While this suffices to realize simple non-reciprocal transport, the overall functionality remains limited and inadequate for supporting more sophisticated topological control.

In contrast, three-level systems—as the simplest multilevel models—exhibit parameter spaces that support higher-order exceptional points and multichannel interference. These features provide substantially greater degrees of freedom for non-Hermitian dynamical manipulation, and have recently emerged as a frontier research focus in the field [68,69,70,71]. Due to its higher state space dimension and multiple coupling channels, it supports richer singularity topologies than two-level systems, such as third-order singularities (EP3) or networks composed of multiple second-order singularities (EP2). At these singularities, the system’s eigenstates and eigenvalues simultaneously degenerate, inducing more complex non-Hermitian dynamical behavior. Compared to two-level systems, three-level systems exhibit more pronounced chiral responses, non-adiabatic transition characteristics, and path-dependent final state selectivity during evolution around singularities. The combination of multiple intrinsic transition channels within the system and quantum interference effects results in highly diverse final state distributions, yielding outcomes far exceeding the binary outputs of two-level systems. These fundamental differences in singularity topology, multi-channel interference, and complex energy spectrum structures mark a qualitative change in dynamical behavior from two-level to three-level systems, laying the foundation for constructing a general theory of multi-level non-Hermitian systems.

At the methodological level, time-dependent perturbation theory serves as a crucial tool for investigating the interaction between a system and external driving forces [72,73]. Extending this theory to non-Hermitian three-level systems not only revolutionizes the theoretical framework of perturbation theory itself but also establishes a direct link between the properties of static singularities and their dynamical responses. This facilitates the revelation of the dynamic evolution of non-Hermitian effects in observable quantities, shifting the research focus from static properties to dynamical processes [74,75]. Investigating the response of three-level systems under time-dependent driving reveals novel physics arising from higher-order singularities, such as the potential enhancement of sensitivity during EP3 evolution. Furthermore, the system’s intrinsic multiple transition channels enable non-reciprocal path manipulation under non-Hermitian control (e.g., selective gain or loss), thereby achieving quantum control functions difficult to realize in conventional Hermitian systems.

Within the framework of time-dependent perturbation theory for Hermitian systems, transition probabilities between quantum states exhibit bilateral symmetry. However, in non-Hermitian systems, quantum transitions can induce chiral flipping between initial and final states, leading to non-commutative transition probabilities that violate conservation laws. This highlights the intrinsic connection between chiral behavior and transition probabilities [76]. Research on non-Hermitian three-level systems in [77] demonstrates that time-dependent non-Hermitian interactions can induce significantly asymmetric transition processes under weak perturbation conditions. The theoretical framework constructed in this study focuses on the dynamics of intrinsic non-Hermitian systems: starting from a stationary Hermitian system, time-dependent non-Hermitian perturbations are introduced. Within this framework, the unperturbed Hamiltonian remains Hermitian, enabling the analytical solution of the evolution of the real-space wave function over the entire time domain. Based on this analytical approach, the study reveals asymmetric transition probabilities arising from non-Hermitian properties. Furthermore, when system parameters traverse different directions within the parameter space, they induce chirality-dependent final state selection behavior. It should be noted that the transient perturbations employed in the literature exhibit a continuous spectrum in the Fourier transform, thereby establishing the foundation for theoretical analysis. However, for systems described by non-Hermitian Hamiltonians, quantum transitions induced by time-dependent perturbations warrant further investigation.

These two papers expand the framework of time-dependent perturbation theory for non-Hermitian systems from different dimensions [73,78]. The first paper addresses static non-Hermitian systems by treating spatial jump terms as perturbations, expanding the time-evolution operator, and analytically solving the time-dependent evolution of wave functions. This reveals the microscopic mechanisms behind novel dynamics such as “edge explosions,” constituting a “time-dependent perturbation theory for time-independent Hamiltonians.” The second paper addresses systems with explicitly time-dependent Hamiltonians. Under the adiabatic approximation, it treats the complex potential as a perturbation. Based on the invariant operator method and standard perturbation formulas, it systematically calculates analytical corrections to the system’s energy (which exhibits non-zero imaginary parts) and wave functions. This falls under the “perturbation theory for instantaneous eigenproblems of time-dependent Hamiltonians.” The fundamental distinction between the two lies in their focus: the former investigates the dynamical response of intrinsically non-Hermitian systems, while the latter examines the instantaneous corrections to time-dependent systems perturbed by complex potentials. Together, they respectively provide two key perturbative frameworks for addressing “non-Hermiticity” and “time-dependence,” systematically advancing the application of perturbation methods in non-Hermitian quantum physics. These approaches constitute complementary and indispensable theoretical tools for understanding and analyzing complex non-Hermitian phenomena.

This paper investigates the time-dependent perturbation dynamics of non-Hermitian quantum systems. To elucidate the regulatory mechanism of time-dependent non-Hermitian perturbations on the transition dynamics of multi-level systems, the biorthogonal basis representation is adopted to study systems composed of an unperturbed static non-Hermitian Hamiltonian and a periodically driven time-dependent perturbation Hamiltonian. Analytical derivations and numerical simulations achieve cross-validation of their results. By integrating non-Hermitian time-dependent perturbation theory with periodic driving mechanisms, this work extends the traditional framework for describing static non-Hermitian systems. It achieves this by introducing periodic non-Hermitian perturbations and applies the expanded theory to the analysis of multi-level systems. Within this framework, the study systematically investigates the dynamic regulation of singular points during parameter variations, providing analytical insights into the corresponding quantum evolution processes. Research indicates that the state transition probability of time-dependent perturbative non-Hermitian systems driven by three-level periodic perturbations exhibits significant asymmetry, specifically a difference between forward and backward transition probabilities. Periodic driving actively amplifies the asymmetry during the transition process. By designing the spectrum of periodic perturbations (e.g., employing a one-sided spectrum), selective manipulation of specific quantum states can be achieved. Within this framework, transition probabilities can be significantly enhanced through resonance, while non-Hermiticity further breaks the inherent symmetry, leading to the phenomenon of “selective resonance” where only transitions in a single direction are significantly amplified. By precisely tuning the drive frequency, coupling between specific channels can be selectively enhanced or suppressed. The amplification of channel asymmetry by non-Hermitian properties ultimately enables highly efficient and selective quantum state excitation.

In Section 2, we introduce a periodically driven three-level non-Hermitian perturbation model. Section 3 derives the dynamical equations for this model analytically. Section 4 confirms through numerical simulations that the dynamical evolution of the three-mode non-Hermitian system agrees with theoretical analytical results, revealing significant chiral transition behavior manifested as non-reciprocal transition probabilities. Finally, Section 5 summarizes the research findings and outlines future directions.

## 2. Three-Level Time-Dependent Perturbation Theory Model

We consider constructing a three-level non-Hermitian quantum system model whose dynamical evolution is described by a time-dependent non-Hermitian Hamiltonian $\hat{H}$ [79]. Furthermore, $\hat{H}$ exhibits symmetry, where $\hat{H}={\hat{H}}^{T}$.

(1) $$\hat{H}=\left(\begin{array}{ccc} ig+\delta & -1 & 0\\ -1 & 0 & -1\\ 0 & -1 & -ig-\delta \end{array}\right)$$

This three-level non-Hermitian model exhibits unique symmetry-induced chiral dynamics, filling a gap in research on three-mode systems. Its physical background lies in non-Hermitian optical systems, where periodic driving and the manipulation of exceptional points enable effective control of chiral transmission. This model features a simple structure and precise symmetry, making it particularly suitable for investigating cutting-edge problems in non-Hermitian dynamics. Not only is it readily implementable on platforms such as optical waveguides [80] and cold atoms [81], but it also demonstrates high experimental feasibility, holding significant promise for advancing related experimental verification.

The Hamiltonian $\hat{H}$ of the system in Equation (1) possesses an explicit time structure, whose mathematical form can be expressed as:

(2) $$\hat{H}\left(t\right)={\hat{H}}_{0}+f\left(t\right){\hat{H}}_{1}$$

In this non-Hermitian system, ${\hat{H}}_{0}$ represents the time-independent static component, while $f\left(t\right){\hat{H}}_{1}$ represents a time-dependent driving perturbation term, which remains consistently much smaller than ${\hat{H}}_{0}$. The time-dependent behavior of the system is described by the complex analytic function $f\left(t\right)$, where ${\hat{H}}_{1}$ is the time-independent perturbation operator.

Therefore, the total Hamiltonian $\hat{H}\left(t\right)$ can be decomposed into the following form:

(3) $${\hat{H}}_{0}=\left(\begin{array}{ccc} ig_{0} & -1 & 0\\ -1 & 0 & -1\\ 0 & -1 & -ig_{0} \end{array}\right)$$

(4) $$f\left(t\right){\hat{H}}_{1}=i\rho e^{i\gamma t}\left(\begin{array}{ccc} -1 & 0 & 0\\ 0 & 0 & 0\\ 0 & 0 & 1 \end{array}\right)$$

The time-dependent behavior of the non-Hermitian Hamiltonian $\hat{H}\left(t\right)$ is dominated by two dimensionless time-varying parameters g and δ. Specifically, it takes the form of $g=g_{0}-\rho \cos\left(\gamma t\right)$ and $\delta =\rho \sin\left(\gamma t\right)$. Therefore, the parameter is defined as such, and its expression can be simplified to $Z\left(t\right)=g_{0}+if\left(t\right)$.

The energy spectrum of the Hamiltonian $\hat{H}$ consists of three discrete eigenvalues. For the Hamiltonian given in Equation (1), the eigenvalues are found to be $\kappa_{0}=0$ and $\kappa_{\pm }=\pm \sqrt{2-Z^{2}}$.

The eigenvalues and eigenstates of the time-unperturbed Hamiltonian (3) can be rigorously determined. When the eigenvalues are $\chi_{0}=0$ and $\chi_{\pm }=\pm \sqrt{2-{g_{0}}^{2}}$, the eigenstates $|\chi_{0}\rangle$ and $|\chi_{\pm }\rangle$ of ${\hat{H}}_{0}$ are given by the expressions:

(5) $$\begin{array}{l} |\chi_{0}\rangle =\frac{1}{\sqrt{n_{0}}}{\left(-1\;\;\;\;-ig_{0}\;\;\;\;1\right)}^{T}\\ |\chi_{\pm }\rangle =\frac{1}{\sqrt{n_{\pm }}}{\left(1-{g_{0}}^{2}\pm ig_{0}\sqrt{2-{g_{0}}^{2}}\;\;\;\;-ig_{0}\mp \sqrt{2-{g_{0}}^{2}}\;\;\;\;1\right)}^{T} \end{array}$$

Here, T denotes transpose, and the normalization constant is given by $n_{0}=n_{\pm }=2-{g_{0}}^{2}$. For a time-independent Hamiltonian ${\hat{H}}_{0}$, its instantaneous eigenvalues and corresponding instantaneous eigenstates do not vary with time.

When the eigenvalues of the Hermitian conjugate Hamiltonian ${{\hat{H}}_{0}}^{†}$ are $\lambda_{0}=0$ and $\lambda_{\pm }=\pm \sqrt{2-{g_{0}}^{2}}$, the corresponding eigenstates are

(6) $$\begin{array}{l} |\lambda_{0}\rangle =\frac{1}{\sqrt{n_{0}}}{\left(-1\;\;\;\;ig_{0}\;\;\;\;1\right)}^{T}\\ |\lambda_{\pm }\rangle =\frac{1}{\sqrt{n_{\pm }}}{\left(1-{g_{0}}^{2}\mp ig_{0}\sqrt{2-{g_{0}}^{2}}\;\;\;\;ig_{0}\mp \sqrt{2-{g_{0}}^{2}}\;\;\;\;1\right)}^{T} \end{array}$$

The left eigenstate $\langle \lambda_{s}|$ is defined as the conjugate transpose of the corresponding right eigenstate $|\lambda_{s}\rangle$, i.e., $\langle \lambda_{s}|={|\lambda_{s}\rangle }^{†}$, which are eigenstates of the Hamiltonian ${{\hat{H}}_{0}}^{†}$. The two sets of eigenstates satisfy a dual orthogonality relation: $\langle \lambda_{s}|\chi_{n}\rangle =\delta_{s,n}$. This relation normalizes a pair of left and right eigenstates with complex conjugate eigenvalues, while exhibiting orthogonality in all other cases. Due to the symmetry of the Hamiltonians, i.e., ${\hat{H}}_{1}={{\hat{H}}_{1}}^{T}$, the components of the left eigenstate $\langle \lambda_{s}|$ coincide with those of the corresponding right eigenstate $|\chi_{n}\rangle$.

**Figure 1:**
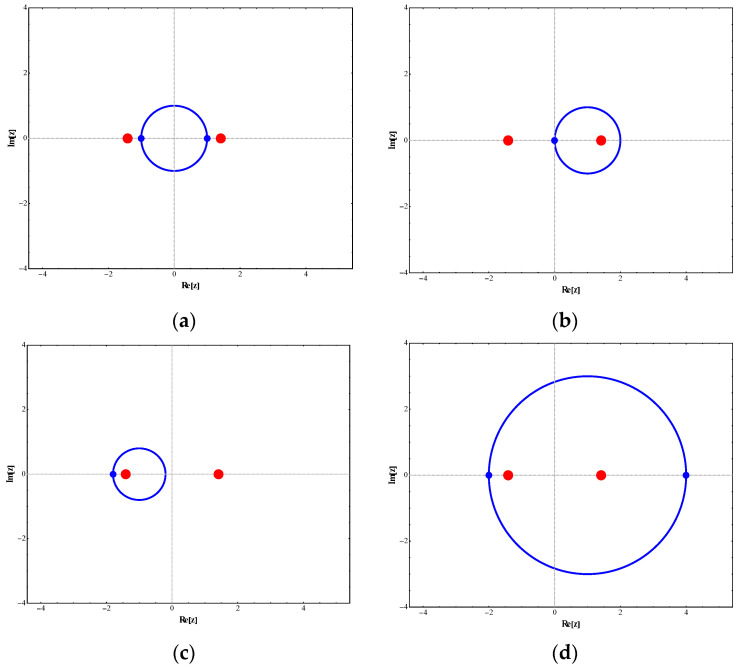
The dynamical loop of the perturbation function in the parameter space. Red points denote exceptional points, while blue points represent initial points.

The key parameters in this three-level model include the non-Hermitian coupling strength g, the energy level detuning δ, the driving amplitude ρ, and the driving angular frequency γ. Their values collectively determine the form of the system’s Hamiltonian and its dynamical evolution path. Here, p characterizes the strength of the periodic driving and acts as a small perturbation. The system is described by the complex parameter $Z\left(t\right)$ in its parameter space, while the driving forms $g=g_{0}-\rho \cos\left(\gamma t\right)$ and $\delta =\rho \sin\left(\gamma t\right)$ form a closed path in the plane centered at $g_{0}$ with radius ρ. When $Z\left(t\right)$ equals $\pm \sqrt{2}$, the system exhibits a third-order singularity, at which point the eigenvalues become completely degenerate ($\kappa_{0}=\kappa_{\pm }=0$). This corresponds to the coordinates $\left(g,\delta \right)=\left(\pm \sqrt{2},0\right)$ of the third-order singularity in the parameter space. The relative magnitude of parameter $g_{0}$ to ρ determines whether closed paths enclose exceptional points and the number enclosed, yielding four scenarios: (1) When $g_{0}=0$ and $\rho <\sqrt{2}$, no exceptional points are enclosed; see Figure 1a. (2) When $0<g_{0}<\sqrt{2}$ and $\sqrt{2}-g_{0}<\rho <\sqrt{2}+g_{0}$, the right exceptional point is enclosed. In this case, the eigenvalues are non-degenerate, so the system does not undergo eigenvalue swapping or eigenstate flipping. see Figure 1b. (3) When $-\sqrt{2}<g_{0}<0$ and $\sqrt{2}+g_{0}<\rho <\sqrt{2}-g_{0}$, the left EP is enclosed; see Figure 1c. (4) When $0<g_{0}<\sqrt{2}$ and $g_{0}+\sqrt{2}<\rho$, both EPs are enclosed; see Figure 1d. The sign of the driving frequency γ controls the direction of the path loop, while the motion period is determined by T=2π/γ, thereby regulating the chiral symmetry evolution of the system. Specifically, when γ>0 is positive, the power loop rotates clockwise; conversely, when γ<0 is negative, the power loop rotates counterclockwise. These parameters collectively characterize the non-Hermitian properties and transition behavior under periodic driving, providing explicit parameter-based insights into the generation mechanism of chiral dynamics in such systems.

## 3. Dynamics of Non-Hermitian Three-Level Systems

In this periodic drive system, time varies from 0 to the period T, and its dynamical behavior is described by the time-dependent Schrödinger equation $i\frac{\partial }{\partial t}\Psi =\hat{H}\Psi$, where ℏ=1 has been chosen.

Let $\Psi ={\left(\psi_{1}\;\;\;\;\psi_{2}\;\;\;\;\psi_{3}\right)}^{T}$ denote the state vector of the system, where the superscript T indicates transposition. The Schrödinger equation satisfied by the quantum state amplitude in this three-level system can be established as follows:

(7) $$i{\dot{\psi }}_{1}=iZ\psi_{1}-\psi_{2}$$

(8) $$i{\dot{\psi }}_{2}=-\psi_{2}-\psi_{3}$$

(9) $$i{\dot{\psi }}_{3}=-\psi_{2}-iZ\psi_{3}$$

Since the introduced set of biorthogonal basis forms a complete set, it can be used to expand any quantum state. The wave function $\Psi \left(t\right)$ is expressed as a linear superposition of these states. $C_{0}\left(t\right)$, $C_{+}\left(t\right)$ and $C_{-}\left(t\right)$ denote the probability amplitudes. Here, $U=\sqrt{2-{g_{0}}^{2}}$.

(10) $$\Psi \left(t\right)=C_{0}\left(t\right)e^{-i\chi_{0}t}|\chi_{0}\rangle +C_{+}\left(t\right)e^{-i\chi_{+}t}|\chi_{+}\rangle +C{\left(t\right)}_{-}e^{-i\chi_{-}t}|\chi_{-}\rangle$$

Solve the Schrödinger equation and substitute into Equation (8). The Schrödinger equations for $C_{0}\left(t\right)$, $C_{+}\left(t\right)$ and $C_{-}\left(t\right)$ are expressed as:

(11) $$i{\dot{C}}_{0}|\chi_{0}\rangle +i{\dot{C}}_{+}e^{-itU}|\chi_{+}\rangle +i{\dot{C}}_{-}e^{itU}|\chi_{-}\rangle =f\left(t\right)C_{0}{\hat{H}}_{1}|\chi_{0}\rangle +f\left(t\right)C_{+}e^{-itU}{\hat{H}}_{1}|\chi_{+}\rangle +f\left(t\right)C_{-}e^{itU}{\hat{H}}_{1}|\chi_{-}\rangle$$

In Equation (9), $U=\sqrt{2-{g_{0}}^{2}}$, where $e^{-i\chi_{n}t}\left(n=0,+,-\right)$ represents the time-evolution phase. Taking the inner product of Equation (9) with the $\langle \lambda_{s}|$ state and utilizing the completeness of the dual orthonormal basis $\langle \lambda_{s}|\chi_{n}\rangle =\delta_{s,n}$, substituting the values of the matrix elements ${\left({\hat{H}}_{1}\right)}_{s,n}$, yields the coupling equations for the probability amplitudes $C_{0}\left(t\right)$, $C_{+}\left(t\right)$ and $C_{-}\left(t\right)$:

(12) $$i{\dot{C}}_{0}=\frac{1}{-U^{2}}e^{-itU}f\left(t\right)\left[C_{+}\left(-2+{g_{0}}^{2}-ig_{0}U\right)+e^{2itU}C_{-}\left(-2+{g_{0}}^{2}+ig_{0}U\right)\right]$$

(13) $$i{\dot{C}}_{+}=\frac{1}{-U^{2}}f\left(t\right)\left[e^{itU}C_{0}\left(-2+{g_{0}}^{2}-ig_{0}U\right)+2C_{+}g_{0}\left(-2g_{0}+{g_{0}}^{3}+iU-i{g_{0}}^{2}U\right)\right]$$

(14) $$i{\dot{C}}_{-}=\frac{1}{-U^{2}}f\left(t\right)\left[f\left(t\right)e^{-itU}C_{0}\left(-2+{g_{0}}^{2}+ig_{0}U\right)+2C_{-}g_{0}\left(-2g_{0}+{g_{0}}^{3}-iU\right)+i{g_{0}}^{2}U\right]$$

The finite-time Fourier integral $I\left(\omega \right)=\int_{0}^{T}f\left(t\right)e^{i\omega t}dt$ of the complex function $f\left(t\right)$ with period T over the interval $\left[0,T\right]$ satisfies the condition that the integral $I\left(\omega \right)$ possesses removable singularities.

Using first-order time-dependent perturbation theory, approximate transition probabilities over one period can be calculated.

(15) $$W_{k,m}\left(T\right)≃{\left|C_{m}\left(T\right)\right|}^{2}\propto {\left|{\left({\hat{H}}_{1}\right)}_{m,k}\right|}^{2}{\left|I\left(\omega_{m}-\omega_{k}\right)\right|}^{2}$$

Based on the above time-dependent perturbation theory, two scenarios are analyzed:

(1) When the parameter γ>0, a pole exists at ω≈−γ<0, indicating that energy is highly concentrated in the negative-frequency region near this frequency. This facilitates transitions when the frequency difference is $\omega_{m}-\omega_{k}<0$ (i.e., $\omega_{m}<\omega_{k}$) in which case transitions occur from higher to lower energy levels. When the frequency difference $\omega_{m}-\omega_{k}>0$ (i.e., $\omega_{m}>\omega_{k}$), transitions from lower to higher energy levels are suppressed. The conclusion is that when γ>0, the perturbation promotes relaxation processes in non-Hermitian systems while suppressing excitation processes.

(2) When the parameter γ<0, the pole is located at $\omega \approx \left|\gamma \right|>0$, which determines that the energy distribution in the frequency domain is highly concentrated near positive frequencies. This distribution characteristic directly influences the properties of the quantum transition process, leading to upward transitions when $\omega_{m}-\omega_{k}>0$ and downward transitions when $\omega_{m}-\omega_{k}<0$. Since the energy in the negative-frequency region is weaker at this point, the transition is significantly reduced. The conclusion is that when γ<0, the perturbation promotes the excitation process of the system and suppresses the relaxation process.

According to Equation (15), the transition probability of the state vector under non-unitary evolution can be calculated for given conditions. Setting t=0, the initial state of the system resides at energy level $|\chi_{0}\rangle$. After undergoing one cycle T of evolution, the transition probability for the system to transition from $|\chi_{0}\rangle$ to $|\chi_{+}\rangle$ is given by Equation (16).

(16) $$W_{0,+}\left(T\right)≃{\left|C_{+}\left(T\right)\right|}^{2}=\frac{8\rho^{2}\;\sin^{2}\left(\frac{k\pi \sqrt{2-{g_{0}}^{2}}}{\gamma }\right)}{\left(2-{g_{0}}^{2}\right){\left(\gamma +\sqrt{2-{g_{0}}^{2}}\right)}^{2}}$$

Set the initial state coefficients to $C_{+}\left(0\right)=1$ and $C_{0}\left(0\right)=C_{-}\left(0\right)=0$, indicating that the system is initially prepared at the energy level $|\chi_{+}\rangle$. Based on this initial condition, the transition probability from one energy level $|\chi_{+}\rangle$ to another $|\chi_{0}\rangle$ within the time interval $\left[0,T\right]$ can be calculated.

(17) $$W_{+,0}\left(T\right)≃{\left|C_{0}\left(T\right)\right|}^{2}=\frac{8\rho^{2}\;\sin^{2}\left(\frac{k\pi \sqrt{2-{g_{0}}^{2}}}{\gamma }\right)}{\left(2-{g_{0}}^{2}\right){\left(\gamma -\sqrt{2-{g_{0}}^{2}}\right)}^{2}}$$

Initialize the system at the energy level $|\chi_{0}\rangle$, with $C_{0}\left(0\right)=1$ and $C_{+}\left(0\right)=C_{-}\left(0\right)=0$. Calculate the transition probability from $|\chi_{0}\rangle$ to $|\chi_{-}\rangle$ resulting from the system’s evolution over the interval $\left[0,T\right]$.

(18) $$W_{0,-}\left(T\right)≃{\left|C_{-}\left(T\right)\right|}^{2}=\frac{8\rho^{2}\;\sin^{2}\left(\frac{k\pi \sqrt{2-{g_{0}}^{2}}}{\gamma }\right)}{\left(2-{g_{0}}^{2}\right){\left(\gamma -\sqrt{2-{g_{0}}^{2}}\right)}^{2}}$$

Set the initial state of the system to energy level $|\chi_{-}\rangle$ (defined by coefficients $C_{-}\left(0\right)=1$ and $C_{0}\left(0\right)=C_{+}\left(0\right)=0$), and then calculate the transition probability from $|\chi_{-}\rangle$ to $|\chi_{0}\rangle$.

(19) $$W_{-,0}\left(T\right)≃{\left|C_{0}\left(T\right)\right|}^{2}=\frac{8\rho^{2}\;\sin^{2}\left(\frac{k\pi \sqrt{2-{g_{0}}^{2}}}{\gamma }\right)}{\left(2-{g_{0}}^{2}\right){\left(\gamma +\sqrt{2-{g_{0}}^{2}}\right)}^{2}}$$

Based on the aforementioned time-dependent non-Hermitian perturbation theory, research indicates that when the finite-time Fourier integral $I\left(\omega \right)$ of an external perturbation exhibits pronounced unilateral spectral characteristics, quantum transitions between energy levels will demonstrate complete unidirectionality. Unless the matrix elements of the Hermitian perturbation are equal, this asymmetry is determined by the finite-time Fourier integral $I\left(\omega \right)$. The parameter γ controls the region of energy concentration. At the same time, the transition probability distribution reveals the direction of the transition, and this probability no longer equals 1. It is worth noting that cyclical drivers further amplify this asymmetry. Accordingly, by designing the duration of perturbations, the evolution direction of non-unitary quantum states can be precisely guided, providing a new theoretical approach for achieving targeted control over their evolution paths and energy transfer.

## 4. Numerical Evolution Simulation

To validate the theoretical results, we subsequently performed numerical simulations of the dynamical evolution equations. Within the framework of non-Hermitian time-dependent perturbation theory, the transition probability following periodic evolution exhibits a pronounced unilaterally asymmetric distribution, specifically $W_{k,m}\neq W_{m,k}$. Notably, periodic driving significantly enhances this asymmetry, as clearly demonstrated in the visualized results of the numerical simulations.

Specifically, when γ>0, the energy of the spectrum $I\left(\omega \right)$ is concentrated in the negative-frequency region $\left(\omega <0\right)$, exhibiting a pronounced peak structure particularly near ω≈−γ. Correspondingly, in the positive-frequency region $\left(\omega >0\right)$, the amplitude of the integral spectrum is strongly suppressed. This directly reflects the PT-symmetry-breaking induced by non-Hermitian properties.

The univalence of this spectrum imposes a strict selection rule on quantum transition processes. Consider a transition between two eigenstates $|k\rangle$ and $|m\rangle$ of the system, with their energy difference denoted as $\Delta \omega =\omega_{m}-\omega_{k}$. When $\omega_{m}>\omega_{k}$, the complex-function integral spectrum energy approaches zero at negative frequencies, it effectively suppresses excitation transitions from lower to higher energy levels. Conversely, for transitions from the higher energy level $|m\rangle$ to the lower energy level $|k\rangle$, when the frequency difference $\omega_{m}<\omega_{k}$, the energy of the integrated spectrum lies in the positive-frequency region, thus yielding: $I\left(\Delta \omega \right)=I\left(\omega_{m}-\omega_{k}\right)\neq 0$. Therefore, the transition probability $W_{k,m}\neq 0$, indicates that transitions from high-energy levels to low-energy levels are permitted and may occur at a high rate.

**Figure 2:**
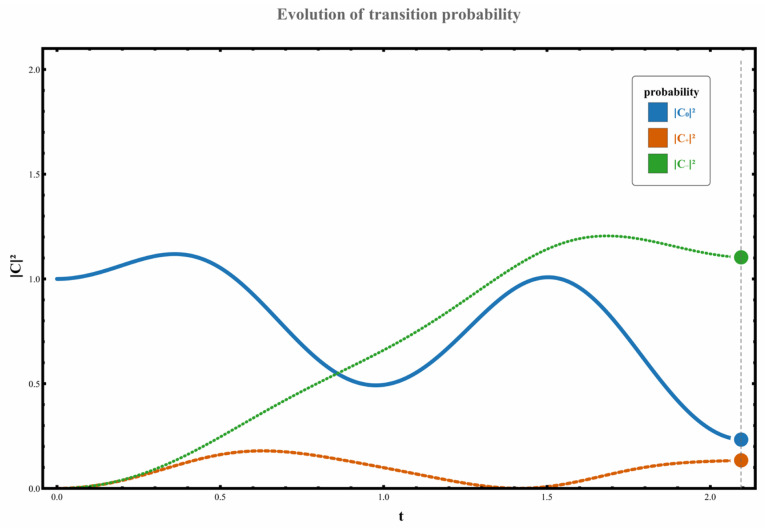
Set parameters to $g_{0}=0$, ρ=1, γ=3; at this point, the dynamical loop does not move clockwise around the exceptional point. The system’s initial state is at $|\chi_{0}\rangle$.

**Figure 3:**
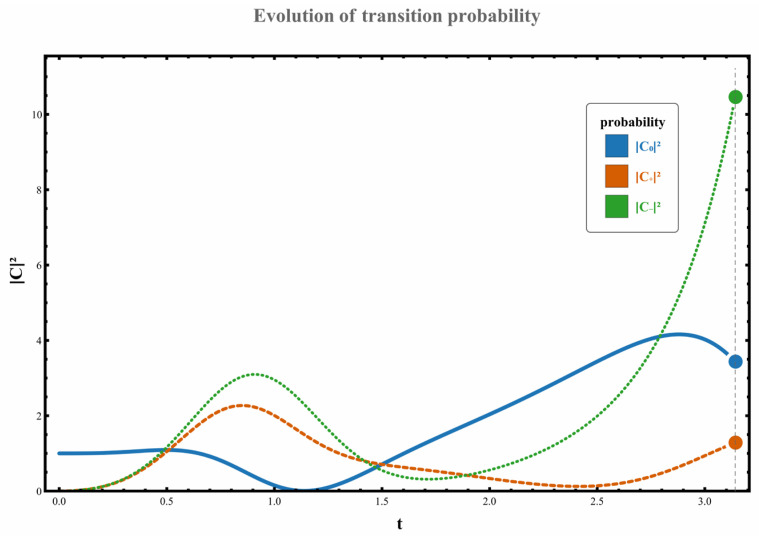
Set parameters to $g_{0}=1$, ρ=1, γ=2; at this point, the dynamical loop moves clockwise around the right exception point. The system’s initial state is at $|\chi_{0}\rangle$, with an initial value of $C_{0}\left(0\right)=1,C_{+}\left(0\right)=C_{-}\left(0\right)=0$.

**Figure 4:**
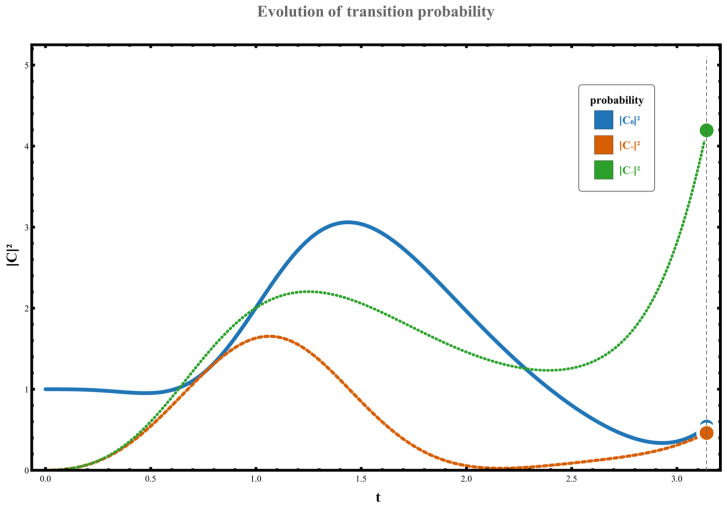
With parameters set to $g_{0}=-1$, ρ=0.8, γ=2; the dynamic loop rotates clockwise around the left exception point, and the system initial state is $|\chi_{0}\rangle$.

**Figure 5:**
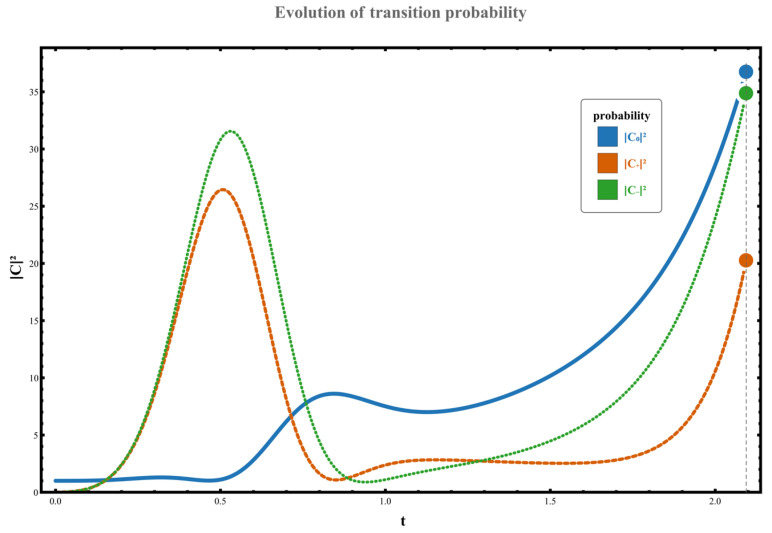
Set parameters $g_{0}=1$, ρ=1, γ=3. Under this configuration, the system dynamics exhibit a clockwise loop containing two exception points, with the system initial state set to $|\chi_{0}\rangle$.

Regarding the dynamic orbital behavior around this three-level exceptional point (EP), the transition characteristics under four distinct orbital paths were analyzed. The specific results are as follows: (1) When the parameter path does not enclose any exceptional points, the system starts from the initial state $|\chi_{0}\rangle$. Although no complete state inversion occurs, the transition probability exhibits significant asymmetry due to the perturbation’s unilateral spectral characteristics (spectral energy concentrated in the negative-frequency region). At this point, the excitation transition probability from state $|\chi_{0}\rangle$ to the higher-energy state $|\chi_{+}\rangle$ is $W_{0,+}≃{\left|C_{+}\left(t\right)\right|}^{2}≃0.133$, while the transition probability to the lower-energy state $|\chi_{-}\rangle$ is $W_{0,-}≃{\left|C_{-}\left(t\right)\right|}^{2}≃1.102$. This behavior, illustrated in Figure 2, demonstrates the asymmetric response in transition direction under non-Hermitian perturbation. (2) When the parameter path encircles the right-hand exception point clockwise, the system undergoes a transition from the initial state $|\chi_{0}\rangle$ to a low-energy state $|\chi_{-}\rangle$, accompanied by a complete state inversion, ultimately occupying state $|\chi_{-}\rangle$. The measured transition probability during this process is $W_{0,-}≃{\left|C_{-}\left(t\right)\right|}^{2}≃10.457$. The specific dynamical evolution is shown in Figure 3. (3) When the path wraps clockwise around the left anomaly point, the system similarly transitions from state $|\chi_{0}\rangle$ to state $|\chi_{-}\rangle$, completing a state reversal and ultimately stabilizing at state $|\chi_{-}\rangle$. The transition probability in this scenario is $W_{0,-}≃{\left|C_{-}\left(t\right)\right|}^{2}≃4.192$, as illustrated in Figure 4, further confirming the influence of encircling direction on transition probabilities. (4) When the path simultaneously encloses two exceptional points, the system still exhibits a transition from a high energy level to a low energy level, flipping from $|\chi_{0}\rangle$ to $|\chi_{-}\rangle$, with the transition probability significantly increasing to $W_{0,-}≃{\left|C_{-}\left(t\right)\right|}^{2}≃37.874$. The corresponding dynamical behavior is shown in Figure 5. In summary, by analyzing transition probabilities and final state selection under different encircling geometries, this study reveals the chiral dynamics of exceptional point encircling in non-Hermitian systems and the regulatory role of spectral unitarity in transition asymmetry.

The results of numerical simulations combined with visualizations indicate that even with different initial states, during clockwise rotation $\left(\gamma >0\right)$, after undergoing one cycle T from the same starting point, transitions from higher energy levels to lower ones are promoted. This confirms the non-reciprocal nature of quantum transition probabilities in non-Hermitian systems, providing crucial evidence for the unidirectionality of transition processes induced by perturbations.

In the study of perturbation dynamics for time-dependent non-Hermitian quantum systems, when the system exhibits net gain, its dynamical behavior displays a high degree of symmetry with the case γ>0, yet possesses significantly different physical properties. At this point, the key analytical properties of the system’s time evolution operator or its associated function $f\left(t\right)$ undergo a reversal, causing the finite Fourier integral $I\left(\omega \right)$ to exhibit once again pronounced unilateral characteristics. In the complex frequency plane, this spectral behavior corresponds to the system parameters traversing a counterclockwise path around the exceptional point. Under the parameter condition γ<0, the energy of $I\left(\omega \right)$ is significantly concentrated in the positive-frequency region $\left(\omega >0\right)$, exhibiting a pronounced peak or energy concentration near $\omega \approx \left|\gamma \right|$. Correspondingly, in the negative-frequency region $\left(\omega <0\right)$, its amplitude is strongly suppressed, meaning the positive-frequency component dominates. This reflects the violation of time-reversal symmetry in non-Hermitian systems. This unilateral reversal of spectral characteristics alters the selection rules for quantum transitions.

Consider the transition between two eigenstates $|k\rangle$ and $|m\rangle$ of the system, with their energy difference denoted as $\Delta \omega =\omega_{m}-\omega_{k}$. When the frequency difference Δω>0, it corresponds to the excitation process from the lower energy state $|k\rangle$ to the higher energy state $|m\rangle$. Since $I\left(\omega \right)$ is non-zero when ω>0 and energy is concentrated, we have $I\left(\omega_{m}-\omega_{k}\right)\neq 0$, corresponding to a transition probability $W_{k,m}\neq 0$ This indicates that the perturbation tends to induce transitions from lower energy states to higher energy states. However, during the transition from the high-energy level $|m\rangle$ to the low-energy level $|k\rangle$, this phenomenon will result in the effective suppression of the jump from higher to lower energy levels due to the weak energy of the spectrum $I\left(\omega \right)$ at a frequency difference of Δω<0.

**Figure 6:**
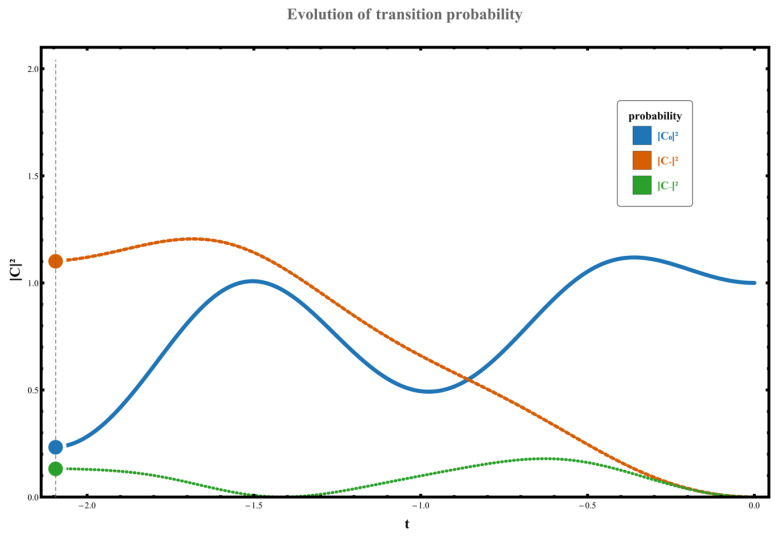
The probability evolution after one cycle of numerical computation is shown in the figure. Here, $g_{0}=0$, ρ=1, γ=−3. The system starts at state $|\chi_{0}\rangle$ and evolves counterclockwise around the exceptional point.

**Figure 7:**
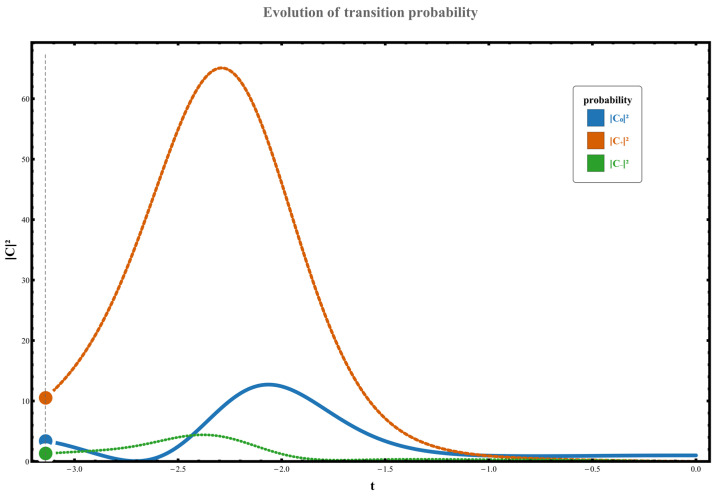
With parameter values $g_{0}=1$, ρ=1 and γ=−2 (where γ<0), the loop rotates counterclockwise around the singular point. The system starts at the state $|\chi_{0}\rangle$, and the numerical probabilities after one cycle of evolution are shown in the figure.

Therefore, when the parameter γ<0, the system exhibits distinct directional selectivity in quantum transitions, effectively promoting one-way transitions from lower energy levels to higher ones. This manifests in the following four scenarios: (1) When the parameter path does not encompass any exception points, the system transitions from the initial state $|\chi_{0}\rangle$ to $|\chi_{+}\rangle$. No state inversion occurs at this point, but due to the unilateral nature of the perturbation spectrum, the system can still undergo an upward transition with a probability of $W_{0,+}≃{\left|C_{+}\left(t\right)\right|}^{2}≃1.602$, as shown in Figure 6. (2) When the parameter path encircles the right-hand exception point counterclockwise, the system transitions from initial state $|\chi_{0}\rangle$ to high-energy state $|\chi_{+}\rangle$, resulting in an eigenstate exchange. The measured transition probability during this process is $W_{0,+}≃{\left|C_{+}\left(t\right)\right|}^{2}≃10.482$. The specific dynamical evolution is shown in Figure 7.

**Figure 8:**
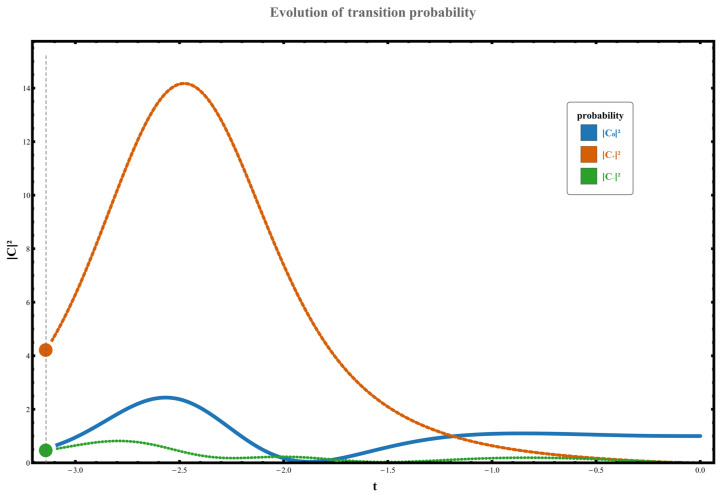
The figure presents the numerical results of the state probabilities after one evolution cycle of the dynamical system. At this point, the loop rotates counterclockwise around the singular point, with initial system values set to $C_{0}\left(0\right)=1$, and $C_{+}\left(0\right)=C_{-}\left(0\right)=0$. Here, $g_{0}=-1$, ρ=0.8 and γ=−2.

**Figure 9:**
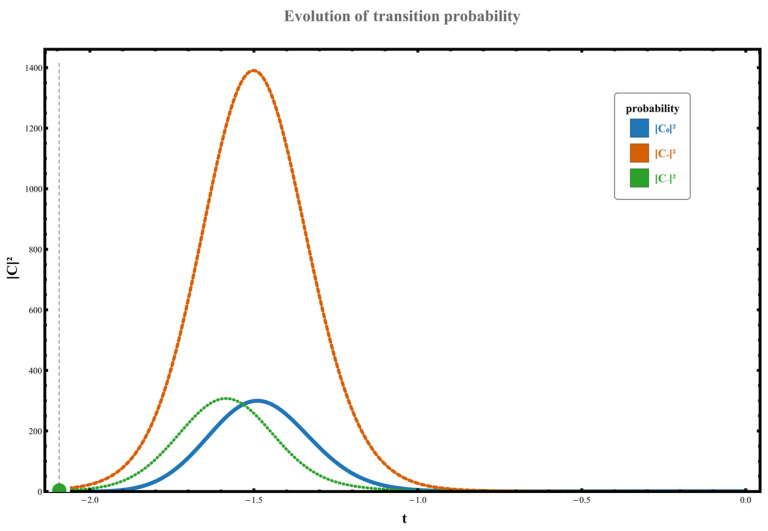
Numerical results for the state probabilities after a single evolution cycle are presented in the figure. At this point, the loop rotates counterclockwise around the singular point, with initial system values set to $C_{0}\left(0\right)=1$, $C_{-}\left(0\right)=C_{+}\left(0\right)=0$. Here, $g_{0}=1$, ρ=3, γ=−3.

(3) When the path encircles the left exception point counterclockwise, the system similarly undergoes an upward transition driven by positive spectral induction, originating from the initial state $|\chi_{0}\rangle$ and ultimately stabilizing at $|\chi_{+}\rangle$. The transition probability in this scenario is $W_{0,+}≃{\left|C_{+}\left(t\right)\right|}^{2}≃4.207$, as shown in Figure 8. The figure presents the numerical results of the state probabilities after one evolution cycle of the dynamical system. At this point, the loop rotates counterclockwise around the singular point, with initial system values set to $C_{0}\left(0\right)=1$, and $C_{+}\left(0\right)=C_{-}\left(0\right)=0$. Here, $g_{0}=-1$, ρ=0.8 and γ=−2, further confirming the influence of encircling direction on transition probability.

(4) When the path simultaneously encompasses two exception points, the system still exhibits a non-Hermitian gain transition, flipping from $|\chi_{0}\rangle$ to $|\chi_{+}\rangle$, with the transition probability significantly increasing to $W_{0,+}≃{\left|C_{+}\left(t\right)\right|}^{2}≃8.228$. The corresponding dynamical behavior is illustrated in Figure 9. Numerical simulations clearly reveal that under periodic driving, the system exhibits asymmetric transition probabilities after evolving one period T from the same initial state. This significant disparity between forward and reverse transition probabilities directly confirms the irreversibility and directional dependence of the transition process in this non-Hermitian system. Following the introduction of periodic driving, the system undergoes continuous non-Hermitian coupling throughout a complete cycle. This dynamical effect markedly amplifies the asymmetry in transition probabilities, rendering the system’s inherent non-Hermitian properties more prominent in observations. This result intuitively demonstrates the unidirectionality and irreversibility of transition channels between non-unitary quantum states, providing unequivocal evidence of symmetry-breaking in transition probabilities.

The agreement between numerical simulations and theoretical predictions validates the effectiveness of non-Hermitian time-dependent perturbation theory and demonstrates that periodic driving can act as an active control tool in open quantum systems, giving rise to dynamical behaviors beyond static frameworks. Through its frequency and phase properties, periodic driving couples with the non-orthogonal eigenstates and complex energy spectrum structure of non-Hermitian systems, thereby enabling the directed manipulation of quantum transition paths and probabilities in the time domain. This process significantly enhances the irreversibility of the system’s evolution. The underlying physical mechanism lies in the periodic drive forcing the system parameters to evolve along closed trajectories in complex parameter space; when encircling exceptional points, the drive imparts distinct chiral features to quantum states, thereby locking in the directionality of transitions. Further analysis indicates that the enhancement of non-Hermitian effects by periodic driving manifests in two respects. First, along specific transition channels, matching the driving frequency to the imaginary-part of the energy-level difference can directionally increase the transition probability of that channel, enabling active selection of the evolution path. Second, periodic modulation reshapes the dependence between dissipation and system parameters; its scaling behavior can be tuned via driving parameters and may exhibit either suppression or staged amplification of nonequilibrium dynamics. Together, these aspects reveal that periodic driving, as an external-field control mechanism, can effectively enhance and steer dynamical behavior in non-Hermitian systems by concurrently influencing transition-path selection and reconfiguring the dissipation relationship.

The dynamical evolution behavior obtained from numerical simulations agrees well with theoretical predictions. Minor deviations attributable to computational precision do not affect the overall trend, which aligns with theoretical expectations. This further supports the pronounced unidirectional evolution between quantum states induced by perturbations under periodic driving. Such an effect amplifies the asymmetry in transition probabilities—which is often subtle in static or transient perturbation settings—and is consistent with the continuous parametric-loop evolution mechanism around exceptional points. By integrating periodic driving with non-Hermitian time-dependent perturbation theory, this study not only confirms the control over transition directions afforded by non-Hermitian coupling and the enhancement of non-Hermitian effects through periodic dynamics, but also establishes, within a general framework, a connection between periodically driven irreversible transitions and chiral behavior associated with encircling exceptional points. This provides a theoretical foundation for probing and utilizing non-Hermitian Floquet physics in platforms such as photonic quantum chips, cold-atom systems, or non-Hermitian optical waveguides. It also lays the groundwork for further analysis of the dynamical properties of Floquet exceptional points, the chiral dependence of transition probabilities, and the long-time evolution of systems toward nonequilibrium steady states.

## 5. Conclusions

In this paper, we investigate perturbation dynamics in time-dependent non-Hermitian systems, focusing on a three-level system. By integrating periodic evolution with time-dependent non-Hermitian perturbation theory, analytical analysis and numerical simulations are mutually validated. The research reveals that non-Hermitian perturbations induce pronounced unidirectional non-reciprocity in quantum transitions, and periodic driving amplifies this asymmetry through cyclic accumulation effects. When energy distributions span positive (negative) frequency regions, the system selectively promotes transitions from low (high) to high (low) energy levels, with non-constant transition probabilities. This asymmetry originates from the system’s gain and loss mechanisms and is intrinsically linked to paths traversing exceptional points, revealing the pivotal role of exceptional points in dynamical control. By integrating periodic driving with non-Hermitian time-dependent perturbation theory, this study employs specific dynamical paths to enhance the intrinsic non-reciprocal behavior of non-Hermitian systems. This approach renders the directional dependence and chiral characteristics of transitions more pronounced and easier to observe. These findings offer novel insights for quantum simulators of non-Hermitian physics, high-sensitivity sensors, and non-reciprocal optical (acoustic) devices.

## Data Availability

The original contributions presented in this study are included in the article. Further inquiries can be directed to the corresponding author.
